# Supplementary material for: COZOID: contact zone identifier for visual analysis of protein-protein interactions
Source: BMC Bioinformatics. 2018 Apr 6;19:125. doi: 10.1186/s12859-018-2113-6 (PMC5889581; doi:10.1186/s12859-018-2113-6)
Supplement: Supplementary file 2 — Software build. Executable binary file of the software tool. (ZIP 118,784 kb) [file 12859_2018_2113_MOESM2_ESM.zip › cozoid/java/docs/beansbinding-1.2.1-doc.zip!/org/jdesktop/el/impl/parser/Token.html]

Token (Beans Binding)


---


|  |  |  |
| --- | --- | --- |
| **Overview** \| **Package** \| **Class** \| **Use** \| **Tree** \| **Deprecated** \| **Index** \| **Help** | |  |
| **PREV CLASS**   **NEXT CLASS** | **FRAMES**    **NO FRAMES**     **All Classes** |  |
| SUMMARY: NESTED \| FIELD \| CONSTR \| METHOD | DETAIL: FIELD \| CONSTR \| METHOD |  |


---


## org.jdesktop.el.impl.parser Class Token

```
java.lang.Object
  org.jdesktop.el.impl.parser.Token
```

---

``` public class Token extends java.lang.Object ```

Describes the input token stream.

---

| **Field Summary** | |
| --- | --- |
| `int` | `beginColumn`             beginLine and beginColumn describe the position of the first character of this token; endLine and endColumn describe the position of the last character of this token. |
| `int` | `beginLine`             beginLine and beginColumn describe the position of the first character of this token; endLine and endColumn describe the position of the last character of this token. |
| `int` | `endColumn`             beginLine and beginColumn describe the position of the first character of this token; endLine and endColumn describe the position of the last character of this token. |
| `int` | `endLine`             beginLine and beginColumn describe the position of the first character of this token; endLine and endColumn describe the position of the last character of this token. |
| `java.lang.String` | `image`             The string image of the token. |
| `int` | `kind`             An integer that describes the kind of this token. |
| `Token` | `next`             A reference to the next regular (non-special) token from the input stream. |
| `Token` | `specialToken`             This field is used to access special tokens that occur prior to this token, but after the immediately preceding regular (non-special) token. |


**Constructor Summary**

`Token()`


| **Method Summary** | |
| --- | --- |
| `static Token` | `newToken(int ofKind)`             Returns a new Token object, by default. |
| `java.lang.String` | `toString()`             Returns the image. |

**Methods inherited from class java.lang.Object**

`clone, equals, finalize, getClass, hashCode, notify, notifyAll, wait, wait, wait`

**Field Detail**

### kind

```
public int kind
```

:   An integer that describes the kind of this token. This numbering
    system is determined by JavaCCParser, and a table of these numbers is
    stored in the file ...Constants.java.

---


### beginLine

```
public int beginLine
```

:   beginLine and beginColumn describe the position of the first character
    of this token; endLine and endColumn describe the position of the
    last character of this token.

---


### beginColumn

```
public int beginColumn
```

:   beginLine and beginColumn describe the position of the first character
    of this token; endLine and endColumn describe the position of the
    last character of this token.

---


### endLine

```
public int endLine
```

:   beginLine and beginColumn describe the position of the first character
    of this token; endLine and endColumn describe the position of the
    last character of this token.

---


### endColumn

```
public int endColumn
```

:   beginLine and beginColumn describe the position of the first character
    of this token; endLine and endColumn describe the position of the
    last character of this token.

---


### image

```
public java.lang.String image
```

:   The string image of the token.

---


### next

```
public Token next
```

:   A reference to the next regular (non-special) token from the input
    stream. If this is the last token from the input stream, or if the
    token manager has not read tokens beyond this one, this field is
    set to null. This is true only if this token is also a regular
    token. Otherwise, see below for a description of the contents of
    this field.

---


### specialToken

```
public Token specialToken
```

:   This field is used to access special tokens that occur prior to this
    token, but after the immediately preceding regular (non-special) token.
    If there are no such special tokens, this field is set to null.
    When there are more than one such special token, this field refers
    to the last of these special tokens, which in turn refers to the next
    previous special token through its specialToken field, and so on
    until the first special token (whose specialToken field is null).
    The next fields of special tokens refer to other special tokens that
    immediately follow it (without an intervening regular token). If there
    is no such token, this field is null.


**Constructor Detail**

### Token

```
public Token()
```


**Method Detail**

### toString

```
public java.lang.String toString()
```

:   Returns the image.

    :   **Overrides:**: `toString` in class `java.lang.Object`

---


### newToken

```
public static final Token newToken(int ofKind)
```

:   Returns a new Token object, by default. However, if you want, you
    can create and return subclass objects based on the value of ofKind.
    Simply add the cases to the switch for all those special cases.
    For example, if you have a subclass of Token called IDToken that
    you want to create if ofKind is ID, simlpy add something like :
    case MyParserConstants.ID : return new IDToken();
    to the following switch statement. Then you can cast matchedToken
    variable to the appropriate type and use it in your lexical actions.


---


|  |  |  |
| --- | --- | --- |
| **Overview** \| **Package** \| **Class** \| **Use** \| **Tree** \| **Deprecated** \| **Index** \| **Help** | |  |
| **PREV CLASS**   **NEXT CLASS** | **FRAMES**    **NO FRAMES**     **All Classes** |  |
| SUMMARY: NESTED \| FIELD \| CONSTR \| METHOD | DETAIL: FIELD \| CONSTR \| METHOD |  |


---
